# A guide for standardized interpretation of lumbar multifidus ultrasonography; an observational study

**DOI:** 10.1186/s12891-022-05590-5

**Published:** 2022-07-16

**Authors:** Remko Soer, Anke Hofste, Frits G. J. Oosterveld, Hermie Hermens, Ricardo van Ravensberg, André P. Wolff, Gerbrand J. Groen

**Affiliations:** 1 Saxion University of Applied Sciences, Research Group Smart Health, Enschede, the Netherlands; 2 University of Groningen, University Medical Center Groningen, Pain Center, Groningen, The Netherlands; 3 University of Groningen, University Medical Center Groningen, Department of Anesthesiology, Groningen, The Netherlands; 4 Roessingh Research and Development, Telemedicine Group, Enschede, the Netherlands; 5 Department of Biomedical Signals & Systems, Faculty of Electrical Engineering, Mathematics and Computer Science, University of Twente, Enschede, Netherlands

**Keywords:** Ultrasound, Electromyography, Interspinal muscles, Erector spinae, Anatomy

## Abstract

**Background:**

Inconsistent descriptions of Lumbar multifidus (LM) morphology were previously identified, especially in research applying ultrasonography (US), hampering its clinical applicability with regard to diagnosis and therapy. The aim of this study is to determine the LM-sonoanatomy by comparing high-resolution reconstructions from a 3-D digital spine compared to standard LM-ultrasonography.

**Methods:**

An observational study was carried out. From three deeply frozen human tissue blocks of the lumbosacral spine, a large series of consecutive photographs at 78 μm interval were acquired and reformatted into 3-D blocks. This enabled the reconstruction of (semi-)oblique cross-sections that could match US-images obtained from a healthy volunteer. Transverse and oblique short-axis views were compared from the most caudal insertion of LM to L1.

**Results:**

Based on the anatomical reconstructions, we could distinguish the LM from the adjacent erector spinae (ES) in the standard US imaging of the lower spine. At the lumbosacral junction, LM is the only dorsal muscle facing the surface. From L5 upwards, the ES progresses from lateral to medial. A clear distinction between deep and superficial LM could not be discerned. We were only able to identify five separate bands between every lumbar spinous processes and the dorsal part of the sacrum in the caudal anatomical cross-sections, but not in the standard US images.

**Conclusion:**

The detailed cross-sectional LM-sonoanatomy and reconstructions facilitate the interpretations of standard LM US-imaging, the position of the separate LM-bands, the details of deep interspinal muscles, and demarcation of the LM versus the ES. Guidelines for electrode positioning in EMG studies should be refined to establish reliable and verifiable findings. For clinical practice, this study can serve as a guide for a better characterisation of LM compared to ES and for a more reliable placement of US-probe in biofeedback.

**Supplementary Information:**

The online version contains supplementary material available at 10.1186/s12891-022-05590-5.

## Introduction

Evidence-based physiotherapy in chronic low back pain (cLBP) has increased exponentially in the last decades [1], reflecting that cLBP should be regarded as a multifactorial disorder, originating or maintained by biological, psychological and social factors [2]. Therapies regarding cLBP should be active in nature and stand-alone passive treatments should be avoided [2]. In general, there is good evidence for the effectiveness of exercise therapies, however their exact mechanisms remain largely unclear and low back pain does not have a reliably identifiable cause that can be defined in terms of purely structural, anatomical or biomechanical aspects [3]. This, however, does not necessarily mean that biological factors are not an important underlying factor for the clinical benefits found after exercise therapies, especially for patients with cLBP. The exercise therapy for these patients has generally included two main streams, i.e., lumbar multifidus (LM) training (‘stabilization’) and general exercise therapies ((cognitive) functional training’) [4]. However, which therapy to choose is not always clear and studies indicate no superiority on group level of one of the treatments [4].

There appears to be lack on valid and consented measurements of LM function and morphology [5]. Most LM studies used electromyography (EMG) [6–8], ultrasonography (US) [9–11], Computer Tomography (CT) [12] or Magnetic Resonance Imaging (MRI) [13]. While CT and MRI provide sufficient detail, the interpretations of the exact borders between LM and adjacent long erector spinae (ES) muscles were found inconsistent [5], as well as interpretations in defining superficial versus deep ‘stabilizing’ muscles [14]. Especially in measurements that can be performed at hand in primary care daily practice (EMG and US), the identification of the borders of LM and subsequently, electrode or transducer placement respectively, appears difficult. While electrode placements have been standardized in the SENIAM project [15], differences in the locations of intramuscular and superficial LM electrodes in EMG studies induced conflicting evidence of ‘true’ or ‘false’ LM activation [14, 16]. A valid positioning of the LM-electrodes is mandatory for the prevention cross-talk signals and for co-activation signals from the adjacent longissimus muscles [17]. Furthermore, in LM-EMG, separate functions were attributed to superficial and deep fibers [17–19]. Finally, a correct demarcation of LM vs ES is important when lumbar spine muscle ultrasonography is used as biofeedback treatment [20, 21].

Because of these discrepancies, there is a need for better standardization of the LM-ultrasonography as a prerequisite for evidence-based physiotherapy The aim of this study is to develop a guide for physiotherapists to better elucidate the sonoanatomy of the LM. We focus upon the so-called superficial, lateral and deep components by comparing high-resolution reconstructions from a 3D digital spine and standard LM ultrasonography.

## Methods

### Design

Observational study.

### Procedures

Three lumbosacral specimens from two human cadavers were obtained. High-resolution photographs of anatomical cross-sections were taken from deeply frozen human tissue blocks (T11-coccygis, female, age 82, BMI 21; and L1-L5 and L5-sacro-coccygeal junction, male, age 40, BMI 24). These specimens were derived from bodies donated to the Dutch nationwide donation program. From these persons written informed consent was obtained during life that allowed the use of their entire bodies for educational and research purposes. From each tissue block, with a heavy-duty sledge cryomicrotome (PMV, LKB Instruments, Stockholm, Sweden) sections were removed and the surface of the block was photographed at 78 μm intervals. A total of 5700 digitized photographs were obtained ranging from 3.0 and 12.6 Mb per photo in size). By multiplanar reformatting, cubes of 3-dimensional tissue pixels (voxels) were reconstructed by self-developed software (Enhanced Multiplanar reformatting Along Curves, E-MAC® [22, 23]. In this manner, images of the three orthogonal planes (sagittal, coronal, and transversal) and oblique cross-sections within the same specimen were obtained. The technique is described in detail elsewhere [24, 25]. The cross-sections used comprised the area between the caudal part of the sacrum and vertebral body L1.

### Ultrasound

Standard ultrasound images were obtained from 1 matched healthy volunteer (male age 39, BMI 22.7) matching the 40 year old specimen, using a 4–17 MHz linear array transducer (E-CUBE11, dynamic, Almelo, The Netherlands), after signing informed consent. Transverse and oblique short-axis views were obtained from the most caudal insertion of LM to L1 with the patients laying on a table with a pillow under the stomach.

### Data analysis

Three authors (RS, AH, GJG) studied the insertion and origin of LM, trajectories and relative position of the LM and deep dorsal musculature at different levels of the lumbar spine, and differences in interpretation were discussed until consensus was reached. Finally, for comparison, the original figures of Macintosh et al. [17] were assembled into one figure displaying the five LM bands that attach to the lateral parts of the spinous processes of L1 to L5 and their mutual topography in cross-sections at L4 and L5. Cross sectional areas and muscle thickness were measured within the E-software program [22, 23] by delineating the muscle-outlines and maximal antero-posterior diameter, subsequent pixel counting revealed the surface area in mm^2^ and diameter in mm. This was done in axial cross-sections.

## Results

**Fig. 1:**
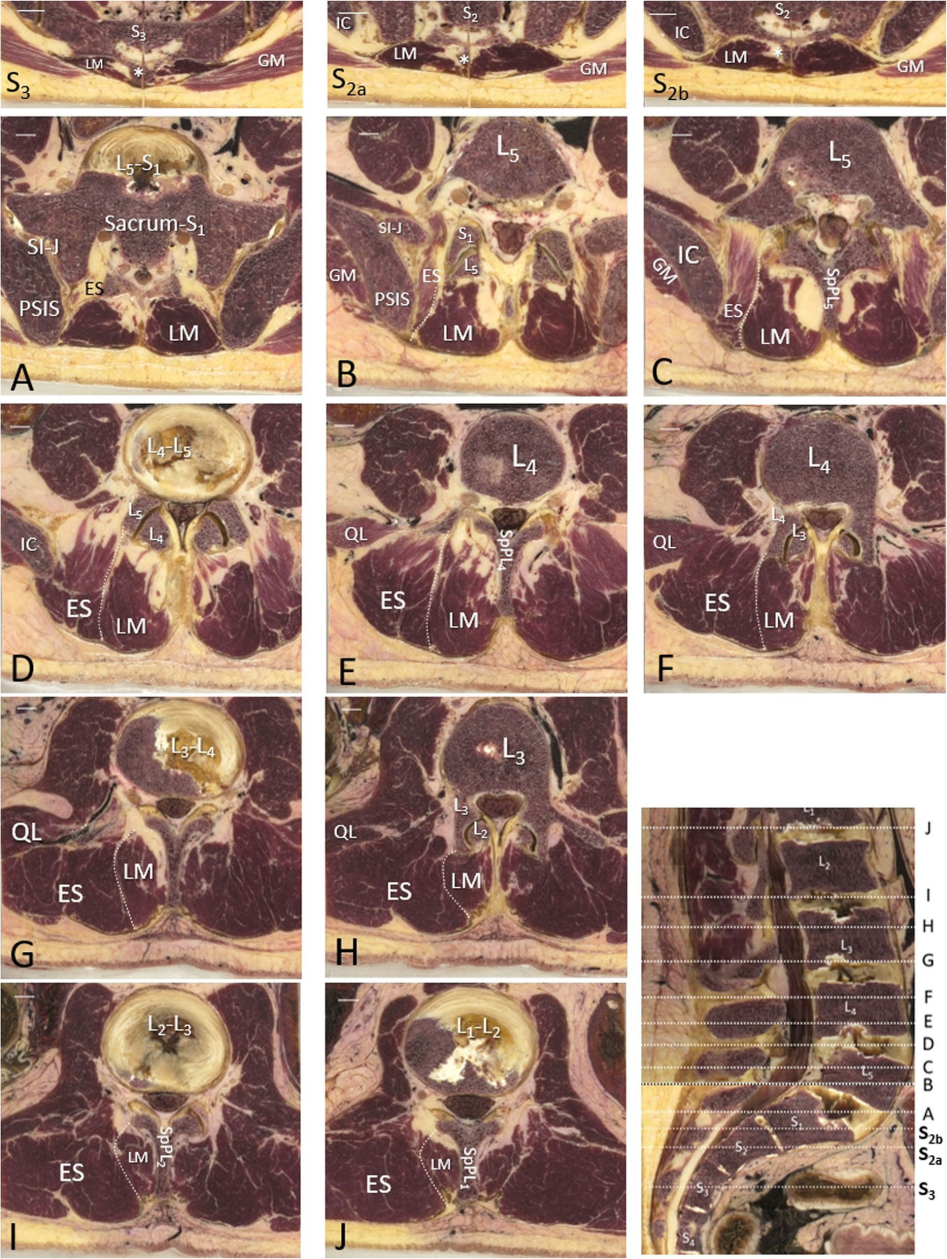
(S3-J). Consecutive transverse cross-sections of the lumbosacral spine perpendicular to the skin from S3 to intervertebral disc L1-2 (see inset at bottom right). From its sacral origin up to the level of the posterior superior iliac spine (PSIS) the lumbar multifidus (LM) is the only dorsal muscle present (Fig. S3-A). At the level of the PSIS lateral to LM the erector spinae (ES) originates from the medial sides of PSIS and adjacent iliac crest (IC), and from the dorsal ligaments of the sacro-iliac joint (SI-J) (Fig. A-C). Cranial to halfway the spinous process of L5 (SpPL5, Fig. C) ES-width is larger than that of LM (Fig. D-J). LM can be detected superficially caudal to the level of intervertebral disc L3-4 (Fig. G), and is deep to the ES cranial to body L3 (Fig. H). GM = Gluteus Maximus; QL = Quadratus Lumborum. S2, S3, L5, L4, L3: vertebral body S2, 3, L3-5; L5/S1, L4/L5, L3/L4 and L2/L3 refer to the facet joints. Bar represents 10 mm

First, the consecutive axial anatomical cross-sections from caudal (level sacrum) to cranial (level body L1) are shown to display the topography (Fig. 1) and size of the LM relative to the ES of the 40 year old specimen (Table 1). From the most caudal LM fibers to approximately the level of the L4/L5 facet joint, the LM demonstrates a higher cross-sectional area (CSA) compared to the ES. From L4-5, the ES increases rapidly in CSA and progressively overlaps LM from lateral to the medial side, to completely overlap LM from L3 towards cranial. The ratio between LM- and ES- CSA approximates 1:1 around L4-5 (Table 1). Fatty tissue was found deep and deep-medial to LM, direct dorsal to the lumbar facet joints and dorsolateral to the lumbar laminae and spinous processes (Fig. 1A-J). In our small series, we observed left–right differences in amount of fatty tissue between L3 and L5 (13–28% left; 7–20% right), in which at each level the left side showed the largest amount.

**Table 1:** Measurements of cross-sectional area and maximal depths of lumbar multifidus and erector spinae

		**LM CSA**	**LM depth**	**LM CSA**	**LM depth**	**ES CSA**	**ES CSA**	
**Level**	**LM**	**L** (mm^2^)	**L** (mm)	**R** (mm^2^)	**R** (mm)	**L** (mm^2)^	**R** (mm^2^)	**Ratio**^**a**^
**PSIS**	**A**	370	21	480	25	-	-	-
**Caudal body L5**	**B**	735	36	651	35	336	254	2:1
**Sp. Proc L5; cranial body L5**	**C**	779	41	824	43	651	611	3:2
**Disc L4-5**	**D**	918	53	918	52	1017	1069	1:1
**Sp proc L4; caudal body L4**	**E**	942	56	967	57	1176	1176	0.8:1
**Sp proc L3-L4; cranial body L4**	**F**	735	43	752	42	1405	1345	1:2
**Sp proc L3; caudal body L3**	**G**	651	48	692	45	1527	1786	1:2.5
**Cranial body L3**	**H**	280	27	322	31	2139	2213	1:8

LM levels A-H reflect figures depicted in Fig. 1

^**a **^Refers to estimated LM:ES ratio; *L* Left, *R* Right, *LM* Lumbar Multifidus, *ES* Erector spinae, *CSA* Cross sectional area, *Sp proc* Spinous Process

### Internal LM topography

**Fig. 2:**
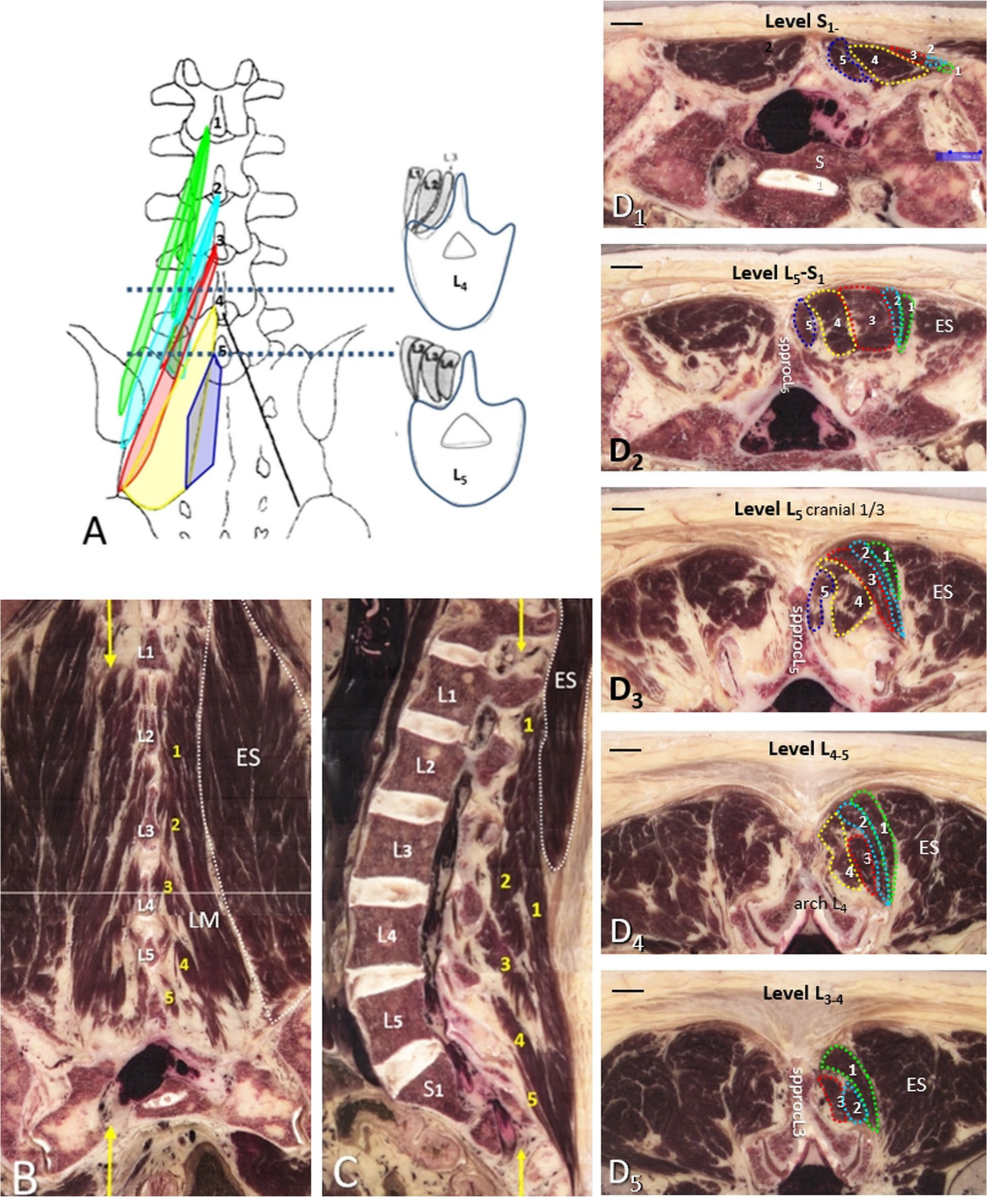
80 year old specimen. **A** Schematic drawing of the assembled multifidus bands attached to the spinous processes of L1-L5 with their relative position in dorsal view and transversal drawings at L4 and L5 based upon the original illustrations of Macintosh et al. [17]. Each color represents a separate band, from medial to lateral, purple (L5), yellow (L4), red (L3), blue (L2) and green (L1), respectively. The transversal cross-sectional drawings show the relative positions of each band at the level of L4 and L5 vertebral bodies. **B, C **position in dorsal view and transversal). The yellow arrows in Fig. B. depict the level of the sagittal plane of Fig.  Coronal (**B**) and sagittal (**C**) reconstructions of the lumbosacral spine at the level of L1-S2 with demarcation line (white dotted line) between the erector spinae (ES) and the separate bands of LM (yellow numbers 1–5). L1-L5 (Fig. **B**), refer to the spinous processes and L1-S1 refer to the vertebral bodies (Fig. **C**, whereas the yellow arrows in Fig. **C** refer to the level of the coronal plane of Fig. **B**. (D1-D5) Consecutive transversal cross-sections from level S1-2 to level L3-4 from the same spine as in Figs. **B** and **C**. The numbers and colored demarcations refer to the LM bands 1–5 depicted in Figs. **A**-**C**. spproc, spinous process; bar represents 10 mm

In the original detailed description of the LM by MacIntosh et al. [17], the LM contained five separate bands connected to the laminae and spinous processes of the five lumbar vertebrae, by which they received their name. For an easier comparison the original figure of MacIntosh et al. [17] was redrawn with all muscular LM bands in one figure (Fig. 2A). In the anatomical cross-sections, an internal configuration of LM with a nearly similar orientation as described earlier could be discerned [17]. The longest bands to the higher lumbar levels (green and light blue in Fig. 2A) are located most laterally, as is shown in the reconstructed coronal cross-section (Fig. 2B), and, depending on the level, as the most superficial bands, until these are covered by ES as shown in the sagittal reconstruction (Fig. 2C). In the transverse plane (Fig. 2D1-5) the mutual orientation at the more caudal levels (up to about L3) predominantly is, from lateral to medial, bands L1, L2, L3, L4 and L5 respectively (Fig. 2D1-4). At higher levels, the orientation of the remaining bands L1-3 becomes more oblique with the L1 band as the most superficial part of the LM (Fig. 2D5). Furthermore, bands L1 and L2 both extend to the deepest parts of the lateral LM (Fig. 2D5). The five LM-bands run more or less parallel to the long axis of the spine and almost perpendicular to the transversal plane (Fig. 2B, C), of which the longest LM bands (L1 and L2) have a somewhat similar orientation as the adjacent ES fibers (Fig. 2B). Throughout the trajectory, at every level the L1 band is closest to the ES (longissimus fibers) (Figs. 2C,  D1- 5). All LM bands could be discerned at the level of the Posterior Superior Iliac Spinae (PSIS), just in between the spinous processes of L5 and S1 (Fig. 2D1). From their caudal attachment (dorsal sacrum; L1 and L2 bands also from the adjacent PSIS (Fig. 2B, C)) to the caudolateral parts of the spinous processes of L1-L5, all bands remain lying adjacent to each other and appear as elongated bands in which superficial and deep parts are continuous with each other, which makes it hard to discern the deep versus superficial LM.

**Fig. 3:**
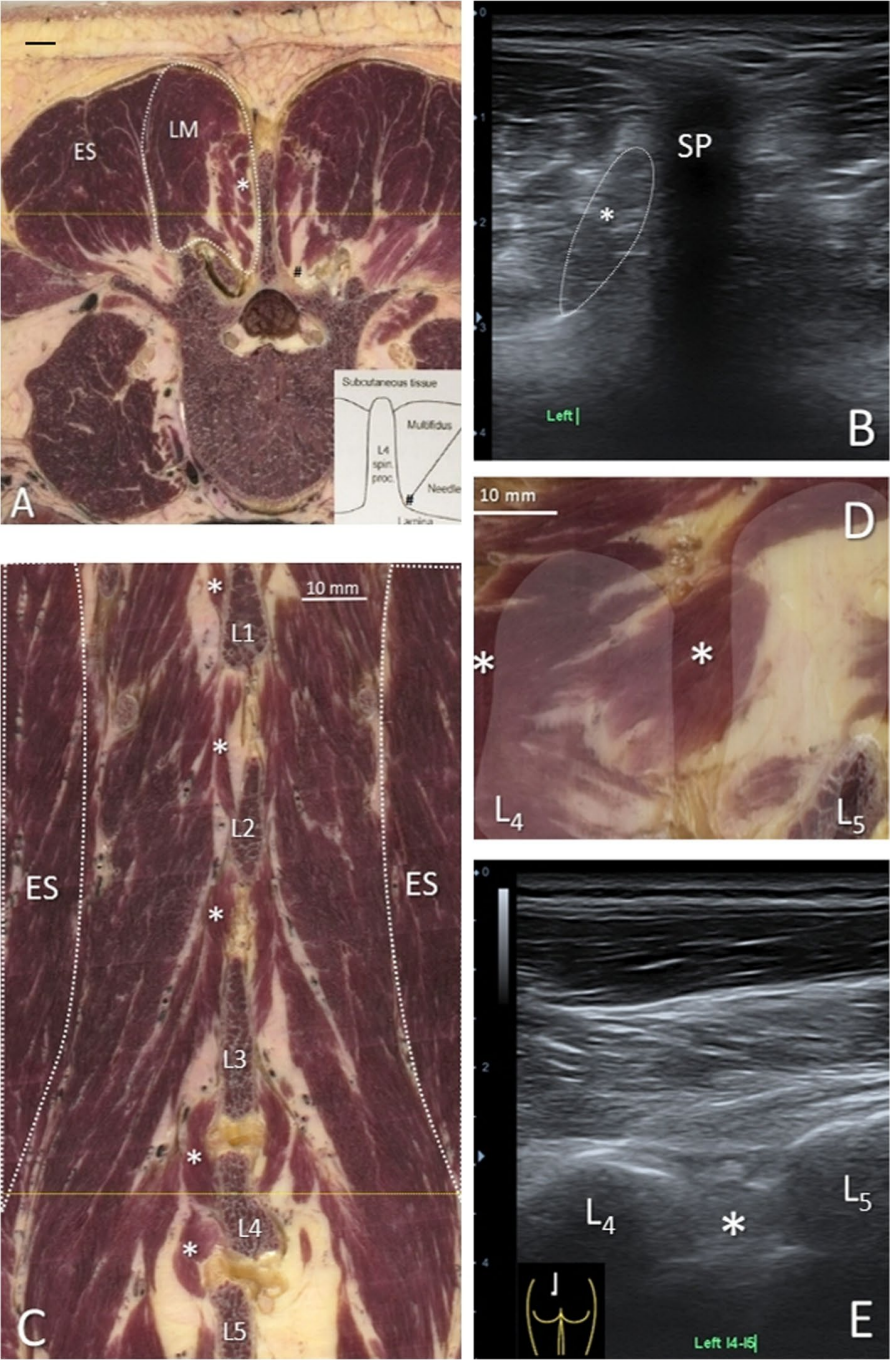
40 year old specimen. Lumbar anatomical cross-sections (figs **A**, **D**), corresponding ultrasonographic (US) views with linear transducer 15 MHz in human volunteer (Figs **B**, **E**) and coronal reconstruction (fig. **C**). (**A**) Transversal cross-section halfway spinous process L4, (see yellow line in Fig. **C**). The erector spinae (ES) lies lateral to LM (dashed contour) and the interspinal muscles (asterisk) are directly adjacent to spinous process L4. The yellow line depicts the location of the plane shown in Fig. **C**. Inset shows the location of a deep LM electrode [19]. (**B**) Detailed transversal US view of LM and interspinal muscles (dashed contour with asterisk at the level of spinous process (SP) L4. (**C**) Reconstruction of coronal plane at the level of spinous processes L1-L5. It shows the oblique lateromedial orientation of LM, of which the most lateral muscles (band L1, 2) appear to have the same orientation as the adjacent ES fibers. Medial to LM, interspinal muscles (asterisks) connect the lateral sides of adjacent spinous processes. In this specimen L4-5 and L5-S1 interspinal muscles are absent at one side (right side). (**D**) Paramedian sagittal reconstruction at level L4-5 showing the adjacent interspinal muscles (asterisks). The contour and location of spinous processes of L4 and L5 is demarcated in white. (**E**) Paramedian sagittal US view of LM at level L4-5, with slightly laterally tilted probe, which enables simultaneous display of interspinal muscles (asterisk) and spinous processes L4-5; inset shows probe positioning

A more detailed view shows that the medial parts of the LM have a close topographical relationship with small muscle fibers that lie medial to these, adjacent to the lateral side of the spinous processes (Fig. 3A in the digital spine (asterisk), and on ultrasound Fig. 3B).

The configuration of these muscle fibers is, however, much more visible from a frontal view (Fig. 3C), by which they can be clearly discerned as interspinal muscles (see asterisks). These short paired muscles attach to contiguous spinous processes and are considered to be the most deep and medial spinal musculature spanning one segment [26].

Also in sagittal reconstructions at this level these interspinal muscles can be identified (Fig. 3D, asterisk), as well as in the corresponding sagittal US image (Fig. 3E). However, in transversal US images the difference between the medial parts of LM and interspinal muscles is not that clear, except for the assumption that every hypoechoic muscular shadow directly lateral to the spinous process should be regarded as an interspinal muscle.

## Caudal configuration of LM

**Fig. 4:**
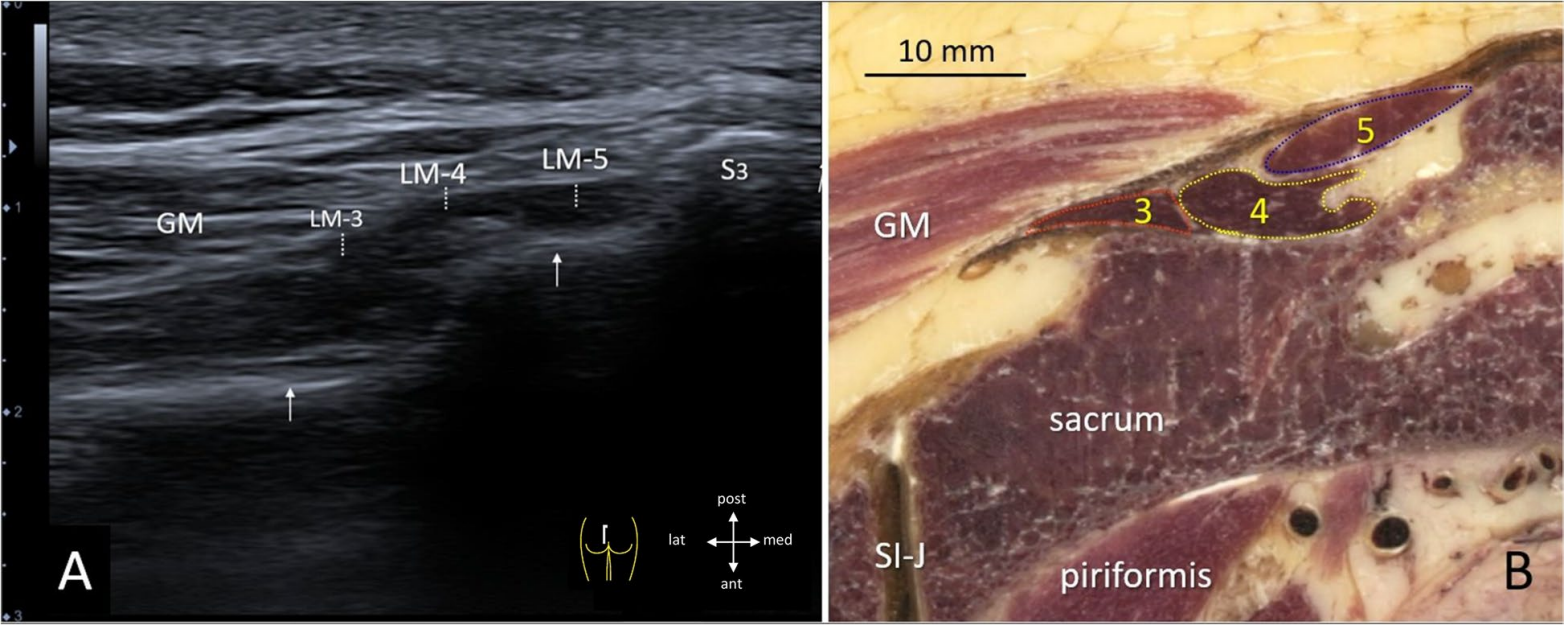
40 year old specimen. **A** Ultrasonographic view with linear transducer 12 MHz in human volunteer of the origin of LM at the dorsal sacrum, level S3 (inset shows position of probe). Arrows indicate the dorsal surface of the sacrum; GM = gluteus maximus. LM 3–5 refer to the separate bands of LM. **B** Transversal cross-section at level S3 with similar orientation as Fig. **A**, showing the three most caudal bands of LM medial and deep to gluteus maximus (GM); each band is demarcated in separate colors; red represents band 3, yellow represents band 4 and blue band 5. SI-J = sacro-iliac joint. I and II refer to the median and lateral sacral crest, respectively. White arrowheads (Figs **A**, **B**) indicate the erector spinae aponeurosis

By ultrasound, the LM configuration as separate bands could be discerned only in more caudal transversal cross-sections (Fig. 4A), especially when similar anatomical cross-sections were used for comparison (Fig. 4B). These caudal bands lie superficial and, finally, run to the spinous processes of L3-5, by which they are labeled accordingly, i.e. LM-3, LM-4 and LM-5. The bands lie just underneath the caudal parts of the erector spinae aponeurosis (ESA), between the median and lateral sacral crest (Fig. 4A, B). Lateral to the LM and superficial to the ESA the insertion of the gluteus maximus can be identified (Fig. 4A,B).

**Fig. 5:**
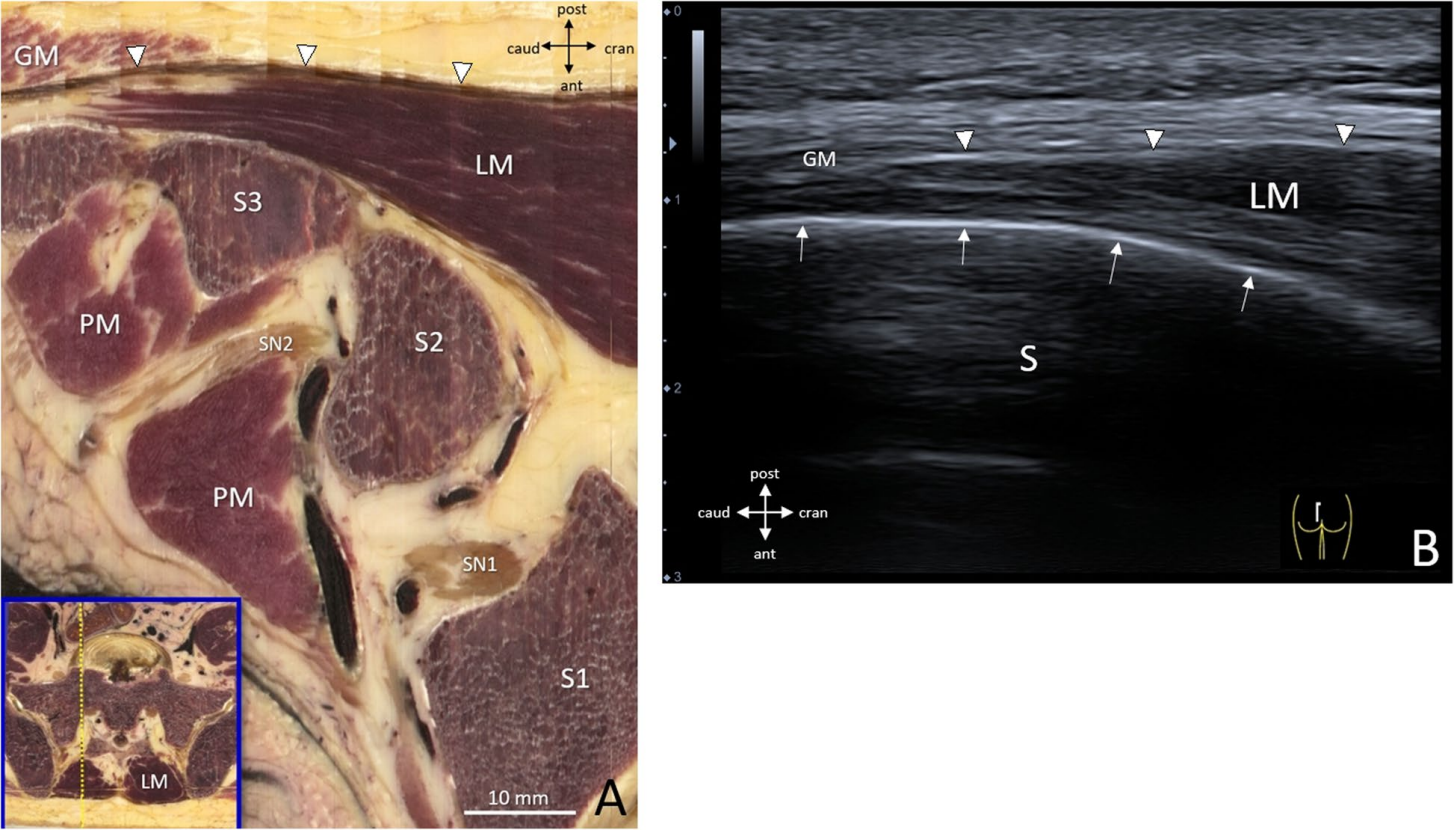
40 year old specimen (**A**) Paramedian sagittal reconstruction of LM origin, 2 cm lateral to the midsagittal plane, as shown in inset. S1-S3, vertebral bodies S1-3; SN1-2, sacral spinal nerves S1-2; LM = Lumbar multifidus; PM = piriformis muscle; GM = gluteus maximus. (**B**) Ultrasonographic view with linear transducer 12 MHz in human volunteer, with probe in similar paramedian sagittal plane (see inset right lower corner). White arrows indicate the dorsal surface of the sacrum (S). White arrowheads (Figs **A**, **B**) indicate the erector spinae aponeurosis (ESA)

In (para)sagittal views, however, the LM shows as a compact muscle in which the separate lumbar bands cannot be discerned. This accounts for both anatomical reconstructions (Fig. 5**A**) and ultrasonographic views (Fig. 5**B**). Located deep compared to the ESA, the most caudal LM fibers insert at the S4 level of the dorsal sacrum, where it is the only muscle present, caudally covered by the most cranial fibers of the gluteus maximus. Muscle thickness increases from 0.5 cm at S3 to approximately 2.0 cm at S1, measured on the digital spine.

**Fig. 6:**
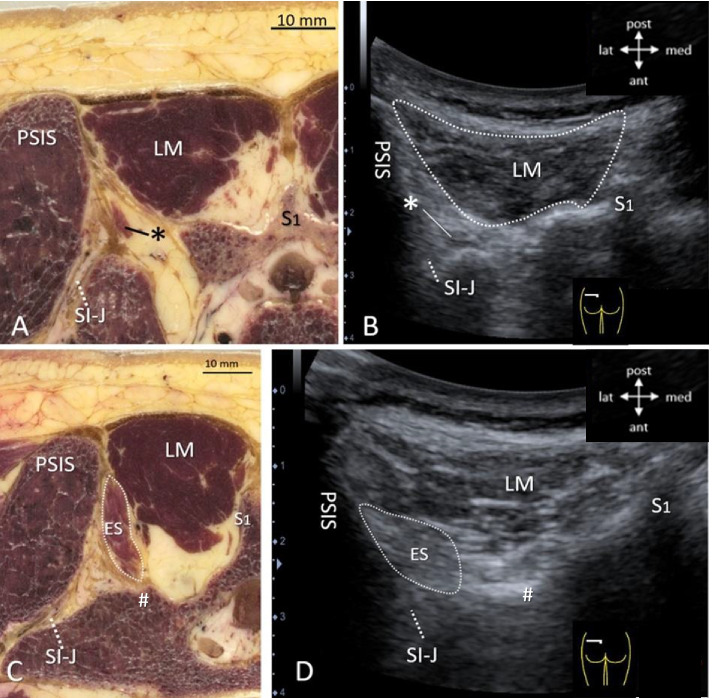
40 year old specimen. **A**-**D**, Transversal cross-sections (**A**, **C**) and matched ultrasonographic (US) views (**B**, **D**; for orientation, see insets) at the level of the posterior superior iliac spine (PSIS) showing a superficial lumbar multifidus (LM) and the origin fibers (*) of erector spinae (ES) lateral and deep to it. ES originates from the PSIS and from the dorsal ligaments of the sacro-iliac joint (SI-J). Figs. **A**, **B** are just caudal to fig. **C**, **D**. (A) S1 = dorsal spine S1. (B) Matched US view with curvilinear transducer 3.6 MHz in human volunteer. At this level, LM is the only superficial muscle (demarcated by white dotted line). Deep to it the origin fibers of ES (*) can be discerned at the dorsal part of the sacro-iliac joint (SI-J) as separate structure with a different echogenic composition compared to LM. (C) S1 = dorsal spine S1. (D) Same curvilinear view as in Fig. **B** showing the different ultrasonographic composition of the ES fibers (demarcated by white dotted line), deep and lateral to LM

At the level of the PSIS, where the LM bypasses the dorsal part of the sacroiliac joint (SI-J), it is no longer the only dorsal muscle, as the erector spinae (ES) appears (Fig. 6 A-D) deep from below and lateral to it. The ES originates from the PSIS, but also to a substantial extent from the dorsal ligaments of the sacroiliac joint (Fig. 6**A**, **B**, asterisk; Fig. **C**, **D**). At this level, the muscle dimensions of the LM are ± 3 cm width × 2 cm depth in the current specimen, and the LM contains all five bands, although they cannot be discerned separately in the axial cross-sections of this specimen (Figs. 6**A**, **C**), which also especially applies to ultrasonographic views (Figs. 6**B**, **D**). In contrast, from its attachment at the dorsal sacroiliac joint ligaments, ES can already be identified by ultrasonography, especially since it displays an echogenic pattern that is different from that of LM, i.e. less hypoechoic (Fig. 6**C**, **D**).

## Discussion

In the present study we have been able to highlight details in standard US LM-imaging that were elusive up to now. Especially since we could compare the US images, generally obtained in non-standard planes, with high-resolution anatomical cross-sections, all reconstructed within the same (digital) specimen, in exactly the same plane.

In this manner we could more easily identify the borders and dimensions of LM and even its separate bands during ultrasonography, however, only in the caudal part of LM. Thus we could discern in the sacral area, from lateral to medial, and more or less in the same superficial layer, bands L3, L4 and L5. More cranial, the LM appeared as a homogeneous hypoechoic mass, less-hypoechoic (i.e. darker) than the adjacent ES fibers.

In the anatomical cross-sections the individual bands could be followed up from the sacral to the high lumbar level using the ‘movie-mode’ of the program in which a series of consecutive cross-sections is displayed in a movie-like manner (see additional material). In the plain cross-sections, however, the mutual location of the individual bands was not always that clear, but basically it has the following pattern from lateral to medial: at S3, bands L3-L5 same layer; at S1, bands L1-L3 same layer but superficial, bands L4-L5 deeper and larger; from L5-S1 upward, all bands L1-L5 adjacent to each other and extending deeply. Since the cranial attachment of each band is a spinous process, the number of bands depends on the lumbar level. Thus, above spinous process L5 only the bands L1-4 are found; above the spinous process L4 only bands L1-3, etc. This latero-medial pattern is more or less similar to that depicted by MacIntosh et al. (see also Fig. 2; [17]), but they did not report on the interspinal muscles, medial to the most medial LM-band.

However, within the LM a superficial-deep pattern in cross-sectional views is hard to discern with the techniques used in the current study.

Consequently, the discrimination between superficial, deep and lateral LM-fibers as described earlier may not be that simple [19]. One should consider, however, that US-guided needle placement may be more easy if comparable anatomical planes are available. Furthermore, we used *undisturbed* anatomy compared to the dissection anatomy of the earlier study [17]. This has the advantage that we can make reconstructions in any plane within the same specimen and perform simultaneous quantitative measurements [27].

In the study by Moseley and colleagues, different EMG activities for deep LM compared to superficial and lateral LM were observed [19]. We were able to mimic the EMG needle approaches as done by Moseley et al., however, as shown in Fig. 3C, those fibers seem to be located about 10 mm lateral of the spinous process, by which the measured activity of ‘deep LM’ might also very well (partly) reflect the nearby interspinal muscles. Also MacDonald et al. discerned deep from superficial LM with separate distinct functions and morphology [28]. They classified LM-fibers crossing just two spinal levels and inserting to the lamina and adjacent articular process and facet joint capsule as deep LM [28]. This could not be confirmed in our study. Furthermore, in our opinion, the needle positions to measure lateral and superficial LM activity used in the study of Moseley et al. [19] appear to be located exactly in the superficial (i.e. longer) parts of medially positioned L3 band and laterally positioned L1-2 band, by which a specific description of lateral vs superficial LM appears superfluous. Moreover, it may be that the needle position to measure deep LM only reflects deep L3-band fiber activity and not concomitantly the activity of deep L1-L2 band fibers, for which the needle should have been placed more laterally at this level.

**Fig. 7:**
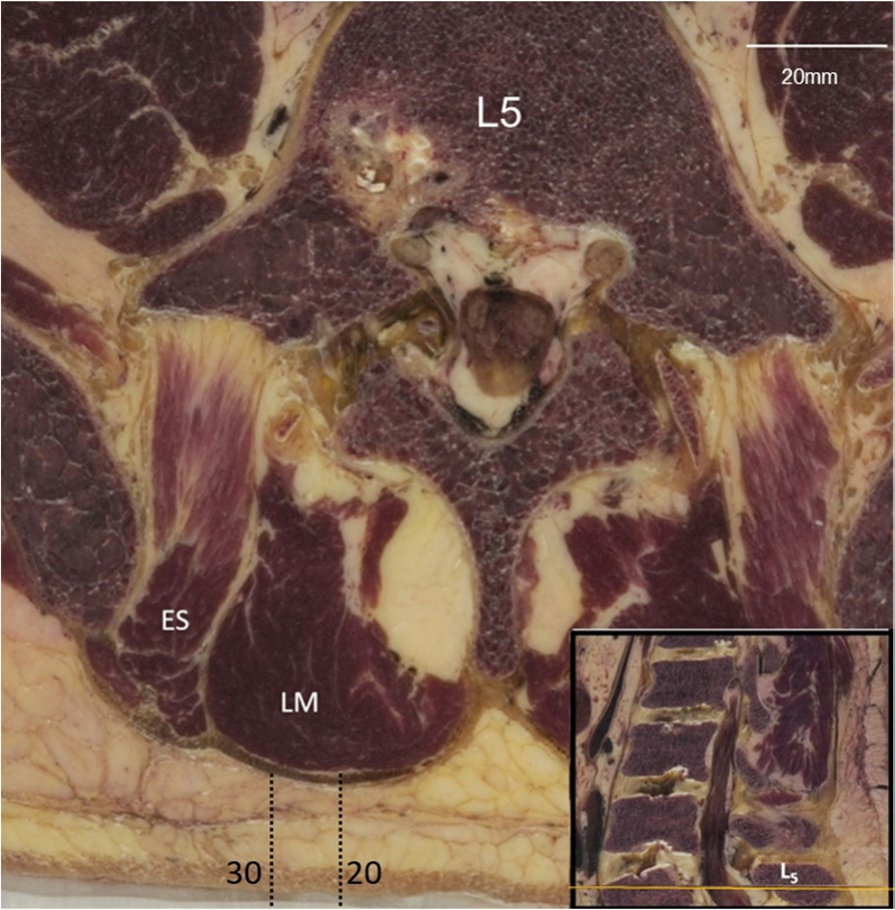
40 year old specimen. Cross-sectional view through mid-spinous process of L5 (see inset); ES = erector spinae; LM = lumbar multifidus. The dotted lines represent the SENIAM guideline for placement of the surface EMG electrode on the skin (between 20–30 mm lateral to the spinous process L5)

Also with regard to surface EMG (sEMG), contradictory results have been reported attributed to differences in electrode placements [14, 16]. Even the SENIAM method, currently the European gold standard for measuring sEMG of LM, advised to place the electrodes at the crossing of a line connecting PSIS and L1 spinous process and a horizontal line through mid-spinous process of L5, at about 2–3 cm lateral from the midline [15]. In Fig. 7, the dotted lines demarcates the 2 to 3 cm distance at L5 for the placement of electrode. As can be seen in Fig. 7, at the same level, most likely LM is being measured, however, cross-talk from adjacent ES fibers cannot be ruled out [29]. A more caudal placement of electrodes closer to the midline may be more preferable. This could affect current guidelines for sEMG-electrode placements in LM studies.

With regard to the close relation between ES and dorsal SI-J ligaments it is tempting to say that the primary diagnostic measures for SI-J pain, i.e. pressure pain in the area of the sacroiliac joint (approximately 3 cm × 10 cm inferior to the ipsilateral PSIS, responding to an intra-articular SI-J local anesthetic block [30, 31] including ≥ 3 positive pain provocation tests (distraction test, compression test, thigh trust test, Patrick sign, Gaenslen test)), suggest that a myofascial origin of SI-J attributed pain may be more important than considered up to now. Very recently it also was put forward that reassessment of the ES muscles would be beneficial to complete the understanding of the attachment sites of these structures in relation to the dorsal SI-J ligament [32]. Surprisingly, our specimens, old as well as young, showed a considerable amount of fatty tissue especially at L4-L5 and L5-S1. This may generally be regarded as fatty atrophy of LM. However, in the young specimen the fatty tissue was predominantly unilateral, and no factors correlated to fatty LM-atrophy, such as disk degeneration, osteoarthritis of facet joints and high BMI were present [33]. This questions if all fatty tissue should be regarded as fatty atrophy, e.g. it has been described in basic anatomy [34]. Moreover, in ultrasonography, the demarcations of LM muscle and fatty tissue are difficult to distinguish, limiting US as a reliable indicator to measure cross-sectional areas of LM. The LM-diameter may be more reliable because of the clear demarcation of the lamina.

Limitations of the study are the low number of specimens (three tissue blocks from two human cadavers), primarily related to the elaborate work to obtain, process and reconstruct the large number of images, and their differing age and gender, which hamper the generalizability of the findings to the general population. Therefore, this study can be regarded as a feasibility study. However, comparing our CSA and fat percentages to previous MRI studies based on healthy subjects and patients with low back pain, we found corresponding results.

In conclusion, the detailed cross-sectional LM anatomy and reconstructions facilitate the interpretations of standard LM US imaging, position of the separate LM-bands, details of deep interspinal muscles, and demarcation of LM versus ES. A clear identification of deep versus superficial versus lateral LM could not be verified. Guidelines for studies using ultrasonography can be developed by using detailed LM-sonoanatomy and should also be taken into account in evidence based physiotherapy for low back pain.

## Supplementary Information

**Additional file 1.**

## Data Availability

The datasets generated and/or analysed during the current study are not publicly available due intellectual property right of G.J. Groen, but are available from the corresponding author on reasonable request.
